# CEBPE expression is an independent prognostic factor for acute myeloid leukemia

**DOI:** 10.1186/s12967-019-1944-x

**Published:** 2019-06-04

**Authors:** Kening Li, Yuxin Du, Dong-Qing Wei, Fang Zhang

**Affiliations:** 1 State Key Laboratory of Microbial Metabolism and School of Life Sciences and Biotechnology, Shanghai Jiao Tong University, Shanghai, 200240 China; 2 State Key Laboratory of Medical Genomics, Shanghai Institute of Hematology, Rui-Jin Hospital, Shanghai JiaoTong University, Shanghai, 200025 China; 3 Key Laboratory of Systems Biomedicine, Shanghai Center for Systems Biomedicine, Shanghai Jiao Tong University, Shanghai, 200240 China

**Keywords:** Acute myeloid leukemia, Survival, Prognostic factors, Relapse, Allogeneic transplantation

## Abstract

**Background:**

Identifying reliable predictive markers is important to make therapeutic decisions, and determine the prognosis for acute myeloid leukemia (AML) patients. However, approximately 50% patients could not be accurately predicted by existing risk factors. It is necessary to identify novel prognostic factors to subdivide the intermediate-risk group or patients without any cytogenetic and molecular abnormalities.

**Methods:**

Kaplan–Meier and Cox regression were used for survival analyses in three independent AML datasets. Analyses integrating both bioinformatics and ChIP-qPCR experiments were performed to explore the role of CEBPE in regulating the expression of known prognostic factors.

**Results:**

CEBPE expression was an independent predictor for both overall survival (OS) and event-free survival (EFS) of AML patients. Moreover, low-expression of CEBPE was found to be associated with high relapse rate. We also proved that differential expression of CEBPE stratified the wild-type patients of multiple genes into good and poor outcomes. In addition, the results showed that no obvious improvement was achieved by allogeneic transplantation in CEBPE high-expressed group, while the survival rate (both OS and EFS) was significantly increased in transplanted patients that with low expression of CEBPE. Finally, we found that CEBPE might regulate the expression of known prognostic factors by localizing on their promoters.

**Conclusion:**

Our findings indicated that CEBPE expression was an independent prognostic factor for AML survival, relapse and allogeneic transplantation, which will provide useful information for outcome prediction and therapeutic decisions.

**Electronic supplementary material:**

The online version of this article (10.1186/s12967-019-1944-x) contains supplementary material, which is available to authorized users.

## Background

Acute myeloid leukemia (AML) is an aggressive malignancy and the most typical leukemia in adults, which is characterized by excessive proliferation, differentiation failure and apoptosis disorder, resulted in the abnormal accumulation of myeloblasts in the bone marrow and peripheral blood [1]. A majority of patients with AML will relapse after achieving complete remission [2]. At present, chemotherapy and/or allogeneic transplantation are the major treatments of AML [3, 4]. According to the acquired cytogenetic and molecular alterations at diagnosis, we could stratify the patients into different prognostic categories, and predict the relapse risk, survival time, drug response and whether a potentially curative allogeneic transplantation is possible [5]. Therefore, identifying reliable predictive markers is important in personalized therapy of AML.

Some risk factors were identified by previous studies, and used to predict treatment outcome for AML patients. For example, patients with genomic translocations such as t(15;17) (lead to PML–RARa fusion protein), t(8;21) (lead to AML1–ETO fusion protein) and inv(16) (lead to CBFb–MYH11 fusion protein) were classified in the favorable-risk group, cytogenetically normal AML (CN-AML) were in the intermediate-risk group, and those with a complex karyotype were classified in the adverse-risk group [6]. Moreover, some molecular abnormalities, including mutations of TP53, CEBPA, FLT3, DNMT3A were also found to provide important prognostic information, especially for CN-AML patients [7, 8]. For example, mutations in CEBPA are associated with a good outcome [9]; internal tandem duplications in FLT3 (FLT3–ITD) adversely affect the clinical outcome [10]. Mutations with prognostic implications in a number of other genes (e.g., TET2 [11], ASXL1 [12, 13], DNMT3A [14], p53 [15] and KIT [12]) have also been identified. To facilitate the prediction of treatment outcome of AML, a standardized system was proposed by an international expert panel in 2010 (working on behalf of the European LeukemiaNet (ELN)) [16]. Based on the published data on the prognostic significance of cytogenetic and molecular alterations, ELN stratified the patients into four groups: favorable, intermediate-I, intermediate-II and adverse. This system refined the classification of AML prognosis [6].

However, the existing risk factors still could not effectively predict the outcome of AML patients for the following reasons. Firstly, approximately 50% patients are CN-AML which is not associated with large chromosomal abnormalities [17]. The relapse rate and survival time of these CN-AML patients are difficult to predict because of high heterogeneity [18]. Secondly, although some gene mutations have statistical significance in predicting survival time of AML (especially for CN-AML), the mutation rates of these genes are relatively low. For example, AML patients with TP53 mutation are predicted to have adverse outcome, but only approximately 5% AML patients are with TP53 mutation [5]. The majority of patients are unpredictable based on gene mutation. Moreover, a significant proportion of patients are classified in intermediate-risk group according to the ELN standardized system [6], but the prognosis of these patients varies, some individuals respond well to chemotherapy based consolidation regimens while others may require allogeneic transplantation. Therefore, it is necessary to identify novel prognostic factors to subdivide the intermediate-risk group or patients without any cytogenetic and molecular abnormalities.

In this study, we found that CEBPE, as a master transcription regulator of myeloid differentiation, was an independent predictor for both overall survival (OS) and event-free survival (EFS) of AML patients. Moreover, CEBPE expression was observed to have prognostic power for AML relapse. Also, CEBPE expression was a potential factor for directing allogeneic transplantation.

## Materials and methods

### Gene expression data of AML patients

We used three independent AML datasets in this study, including The Cancer Genome Atlas (TCGA), GSE1159 and GSE10358. Only samples with both gene expression data and clinical annotations were kept. RNA-Seq data of 184 clinically annotated adult cases of AML were downloaded from TCGA [5]. Microarray data of 260 AML patients were downloaded from GSE1159 [19, 20]. And microarray data of 91 AML patients were downloaded from GSE10358 [21]. Microarray data and cytogenetic risk of each sample in GSE14468 [22, 23] were also used in this study.

### Cell culture

The AML cell lines NB4 and Kasumi-1 were obtained from the American Type Culture Collection (ATCC; Manassas, VA, USA), and cultured in RPMI 1640 medium (Thermo Fisher Scientific, Waltham, MA, USA), supplemented with 10% heat-inactivated fetal bovine serum (GIBCO-BRL), 100 U/mL penicillin and 100 mg/mL streptomycin (GIBCO-BRL). All cells were incubated in a humified 5% CO_2_ at 37  °C.

### Chromatin immunoprecipitation (ChIP) assay

ChIP assay of NB4 and Kasumi-1 cells was conducted by the manufacturer’s Active Motif protocol. Chromatin extracts were immunoprecipitated with anti-CEBPE (Santa Cruz Biotechnology, sc-158) and rabbit IgG (Abcam, ab172730) was used as negative control antibodies. ChIP-qPCR was conducted to analyze immunoprecipitated DNA using SYBR Green PCR Master Mix (Toyobo, Osaka, Japan) and the ABI Prism 7900HT detection system (Thermo Fisher Scientific). Fold enrichment of ChIP DNA vs. input DNA was calculated. The primers were designed to cover regions that are shown in Additional file 1: Table S1.

### Statistical analyses

Survival was estimated according to the Kaplan–Meier method. The log-rank test was used to assess statistical significance. Cox regression was used to assess the association of a given variable with OS or EFS. Multivariable testing was performed using Cox proportional hazards models. P values < 0.05 were considered statistically significant. All of the statistical analyses were conducted using R package “Survival”.

## Results

### CEBPE is actively expressed in AML patients with favorable outcome

**Fig. 1:**
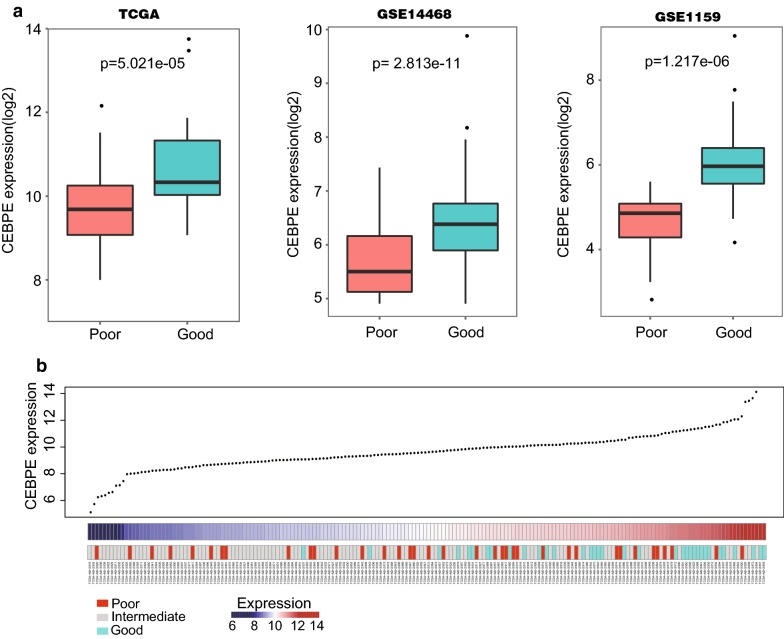
CEBPE expression in AML patients with different prognosis. **a** CEBPE expression of AML patients with good and poor outcomes in three independent datasets TCGA, GSE14468 and GSE1159. **b** CEBPE expression and prognosis classification based on cytogenetic factors in TCGA database

We collected AML gene expression data from TCGA, GSE14468 and GSE1159. The three independent datasets contained 184, 186 and 260 samples, respectively. The information of prognosis classification based on cytogenetic factors was also obtained. The results showed that CEBPE was highly expressed in patients with good prognosis. And this observation was confirmed in all of the three independent datasets. The t-test P-values of CEBPE differential expression between good and poor patients were 5.021e−05, 2.813e−11, 1.217e−6, respectively (Fig. 1a). Moreover, we also found that patients with high expression of CEBPE tended to have good prognosis in TCGA datasets (Fig. 1b).

### CEBPE expression is an independent predictor of AML

**Fig. 2:**
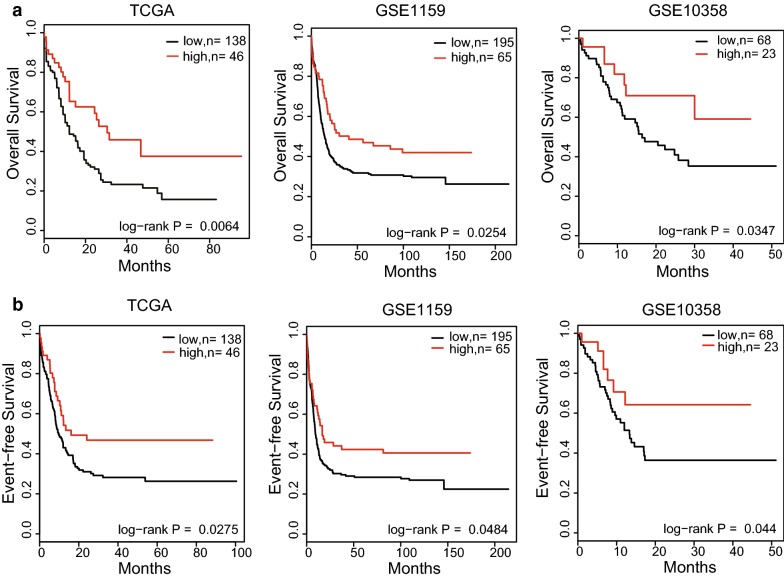
Survival analyses of AML patients with differential expression of CEBPE. **a** Overall survival (OS) analyses of three independent datasets TCGA, GSE1159, GSE10358. **b** Event-free survival (EFS) analyses of three independent datasets TCGA, GSE1159, GSE10358

We then validated the prognostic impact of CEBPE expression in three independent AML datasets, namely TCGA (n = 184), GSE1159 (n = 260) and GSE10358 (n = 91). In each dataset, we ranked the samples according to CEBPE expression, and samples of the top quartile were classified in high-expressed group, while others were classified in low-expressed group. As expected, Kaplan–Meier survival analyses demonstrated that decreased expression value of CEBPE was significantly (P < 0.05) associated with shorter OS and EFS (Fig. 2). In datasets of TCGA, GSE1159 and GSE10358, the 5-year overall survival rates were 38%, 47% and 59% in CEBPE high-expressed group, while 17%, 29% and 35% in CEBPE low-expressed group. Significant difference was also observed in OFS analysis.

In addition, univariable Cox regression analysis demonstrated that patients with higher CEBPE expression showed lower risk. The following variables were evaluated in univariable Cox regression models for outcome: CEBPE expression, age, sex, white blood cell (WBC), peripheral blood (PB) or bone marrow (BM) blasts, the presence or absence of various chromosomal translocations [i.e., inv(16), t(8;21), t(15;17), t(9;11), t(11q23) and t(9;22)] and other abnormalities [+8, −3/inv(3)/t(3;3), −7/del(7q), −5/del(5)], and the presence or absence of gene mutations (FLT3-ITD or FLT3-TKD, DNMT3A, IDH1, IDH2, RUNX1, TET2, TP53, NRAS, CEBPA, KRAS, NPM1, KIT, PHF6 and ASXL1). Variables for which P < 0.1 in univariable analysis were shown in the Table 1 (OS) and Table 2 (EFS). Hazard ratios (HR) > 1 or < 1 indicate, respectively, a higher or lower risk of an event for higher values of continuous variables or for the first category listed for categorical variables in OS or EFS models. Accordingly, we found that age, TP53 mutation, DNMT3A mutation, WBC, t(9;11), RUNX1 mutation were risk factors, while CEBPE expression, t(15;17) and inv(16) were protective factors for AML OS and EFS. Through multivariable testing, we showed that the CEBPE low-expression remained significantly associated with worse OS and EFS in TCGA datasets, after adjusting for all other variables that had P < 0.1 in univariable analyses. Variables for which P < 0.05 in multivariable models were also shown in the Table 3 (OS) and Table 4 (EFS). It turned out that age, TP53 mutation, WBC and CEBPE expression were independent predictors for AML OS and EFS.

**Table 1:** Univariable analyses of overall survival (OS) of AML patients from TCGA database

Variable	HR	95% CI	P-value
CEBPE, high vs. low	0.5	0.3–0.7	0.00029
Age, ≥ 60 vs. < 60	3.2	2.2–4.6	9.40E−10
log2 (WBC), each 2-unit increase	1.2	1.0–1.4	0.040
FLT3, FLT3-ITD vs. others	1.4	0.9–2.1	0.094
NPM1, mutation vs. wild-type	1.4	1.0–2.1	0.071
DNMT3A, mutation vs. wild-type	1.8	1.2–2.6	0.0049
RUNX1, mutation vs. wild-type	1.7	1.0–2.9	0.063
TP53, mutation vs. wild-type	3.4	1.9–5.9	1.98E−05
inv(16) vs. others	0.3	0.1–0.9	0.032
t(15;17) vs. others	0.3	0.1–0.7	0.0075
t(9;11) vs. others	4.1	1.0–16.9	0.052
t(9;22) vs. others	3.4	0.8–14.1	0.086
del (3) vs. others	2.0	0.8–4.1	0.097

*HR* hazard ratio, *95% CI* 95% confidence interval, *WBC* white blood cell, *ITD* internal tandem duplication

Variables for which P < 0.1 in univariable models were shown

**Table 2:** Univariable analyses of event-free survival (EFS) of AML patients from TCGA database

Variable	HR	95% CI	P-value
CEBPE, high vs. low	0.5	0.4–0.8	0.00098
Age, ≥ 60 vs. < 60	2.8	2.0–4.1	2.86E−08
log2(WBC), each 2-unit increase	1.2	1.0–1.3	0.074
DNMT3A, mutation vs. wild-type	1.5	1.0–2.2	0.039
RUNX1, mutation vs. wild-type	1.6	1.0–2.7	0.093
TP53, mutation vs. wild-type	3.2	1.9–5.6	2.76E−05
inv(16) vs. others	0.3	0.1–0.9	0.039
t(15;17) vs. others	0.3	0.1–0.8	0.015
t(9;11) vs. others	3.9	0.9–16.0	0.061
t(11q23) vs. others	2.5	1.1–5.7	0.033

*HR* hazard ratio, *95% CI* 95% confidence interval, *WBC* white blood cell

Variables for which P < 0.1 in univariable models were shown

**Table 3:** Multivariable analyses of OS of AML patients from TCGA database

Variable	HR	95% CI	P-value
CEBPE, high vs. low	0.6	0.4–0.9	0.034
Age, ≥ 60 vs. < 60	2.9	1.9–4.3	3.14E−07
log2(WBC), each 2-unit increase	1.4	1.1–1.7	0.0025
TP53, mutation vs. wild-type	4.5	2.2–9.4	6.22E−05
t(9;11) vs. others	8.4	1.9–36.9	0.0051

*HR* hazard ratio, *95% CI* 95% confidence interval; *WBC* white blood cell

Variables for which P < 0.05 in multivariable models were shown

**Table 4:** Multivariable analyses of EFS of AML patients from TCGA database

Variable	HR	95% CI	P-value
CEBPE, high vs. low	0.6	0.4–0.9	0.042
Age, ≥ 60 vs. < 60	2.6	1.7–3.8	2.42E−06
log2(WBC), each 2-unit increase	1.4	1.2–1.7	0.00056
TP53, mutation vs. wild-type	3.5	1.8–6.9	0.00025

*HR* hazard ratio, *95% CI* 95% confidence interval, *WBC* white blood cell

Variables for which P < 0.05 in multivariable models were shown

### Low-expression of CEBPE predicts high relapse rate

**Fig. 3:**
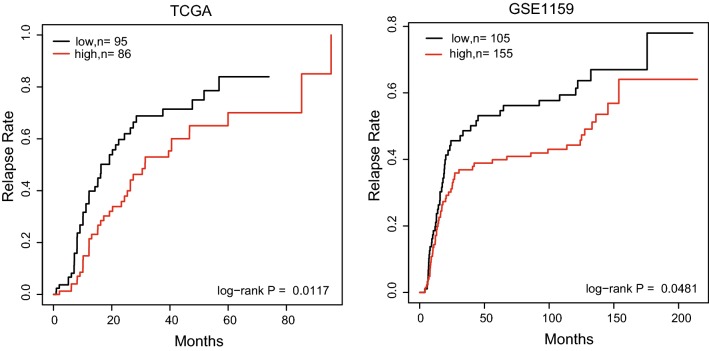
Kaplan-Meier analyses of AML relapse rates after complete remission in TCGA and GSE1159 datasets

We evaluated the association between CEBPE expression and relapse rates after complete remission using datasets of TCGA and GSE1159, which contained the information of relapse. All of the samples were classified into CEBPE high-expressed and low-expressed groups based on k-Nearest Neighbor (KNN) approach. The results showed that CEBPE expression had significant predictive power for AML relapse (P < 0.05). Low expression of CEBPE resulted in an increased incidence of relapse (Fig. 3).

### CEBPE expression has prognostic significance for wild-type AML patients of multiple genes

**Fig. 4:**
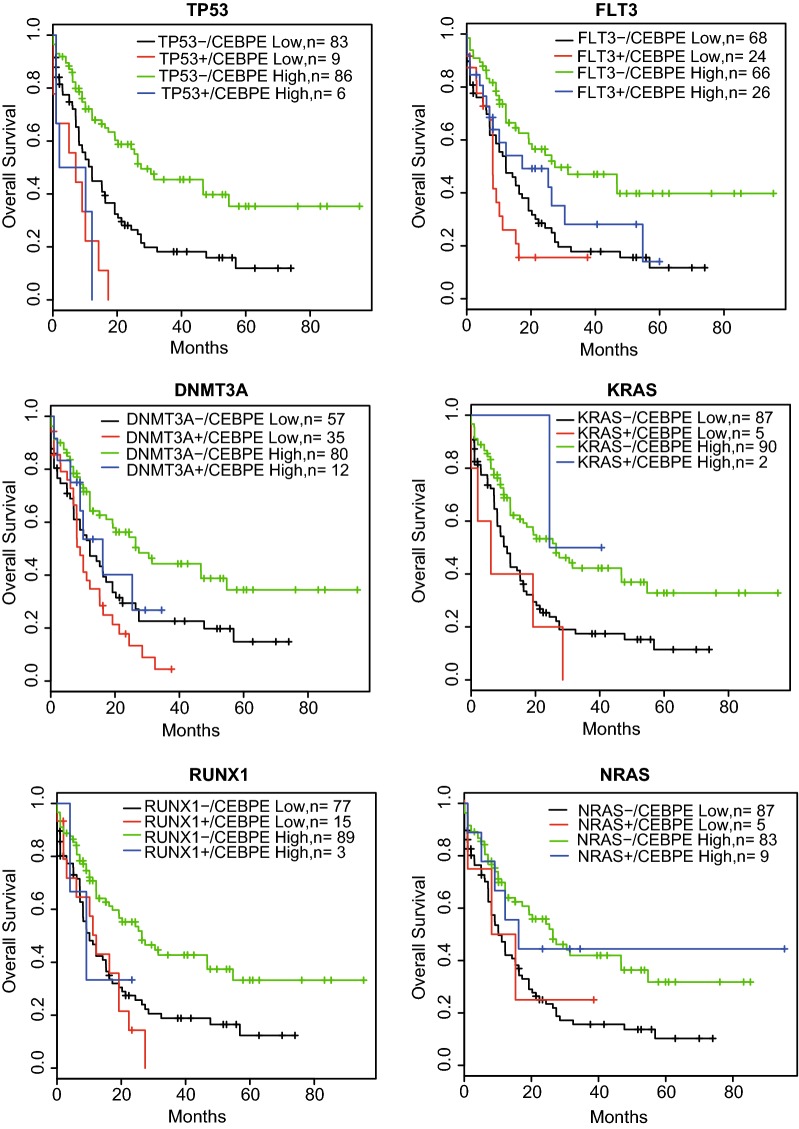
CEBPE expression has prognostic significance for wild-type patients of multiple genes. “+” indicates mutation and “−” indicates wild-type. Differential expression of CEBPE stratified the wild-type patients into good and poor outcomes

Some gene mutations were reported to be associated with poor outcome of AML, such as mutations of TP53 [24], FLT3 [25], DNMT3A [26], RUNX1 [27]. However, the frequency of patients with these mutations was relatively low. Novel prognostic factors were required to predict the outcome of wild-type patients. We evaluated the prognostic power of CEBPE expression for AML wild-type patients in TCGA datasets. For each gene mutation, samples were divided into four classes, namely mutated/CEBPE high, mutated/CEBPE low, wild-type/CEBPE high, wild-type/CEBPE low. The results showed that CEBPE expression differences in wild-type patients of TP53, FLT3, DNMT3A, KRAS, RUNX1 and NRAS were strongly associated with survival time (Fig. 4). Wild-type patients with high-expression of CEBPE showed longer survival than low-expressed wild-type patients. Thus, CEBPE expression could provide useful prognosis information by subdividing the wild-type patients.

### CEBPE expression was a potential prognostic factor for allogeneic transplantation

**Fig. 5:**
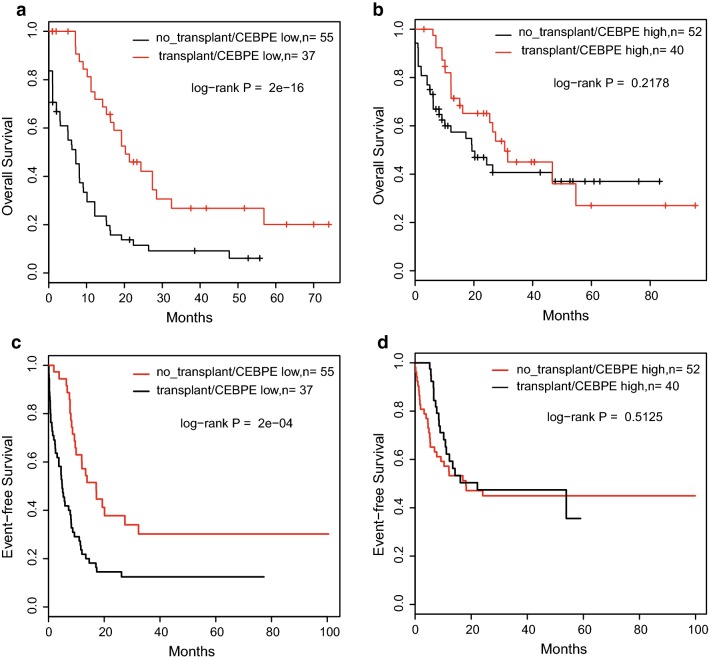
CEBPE expression was a potential prognostic factor for allogeneic transplantation. **a** Overall survival analyses for CEBPE low-expressed patients received or not received allogeneic transplantation. **b** Overall survival analyses for CEBPE high-expressed patients received or not received allogeneic transplantation. **c** Event-free survival analyses for CEBPE low-expressed patients received or not received allogeneic transplantation. **d** Event-free survival analyses for CEBPE high-expressed patients received or not received allogeneic transplantation

We analyzed the association between CEBPE expression and allogeneic transplantation to explore whether CEBPE expression could provide useful information for directing allogeneic transplantation. All samples were classified into CEBPE high-expressed and low-expressed groups based on KNN approach. Then, in each group, Kaplan–Meier survival analyses were applied to compare the survival difference between individuals received and not received transplants. The results showed that no obvious improvement was achieved by allogeneic transplantation in CEBPE high-expressed group, while the survival rate (both OS and EFS) was significantly increased in transplanted patients that with low expression of CEBPE (Fig. 5). These results suggested that CEBPE expression would be a potential predictor for outcome of allogeneic transplantation in AML patients.

### CEBPE regulates known predictors of AML

**Fig. 6:**
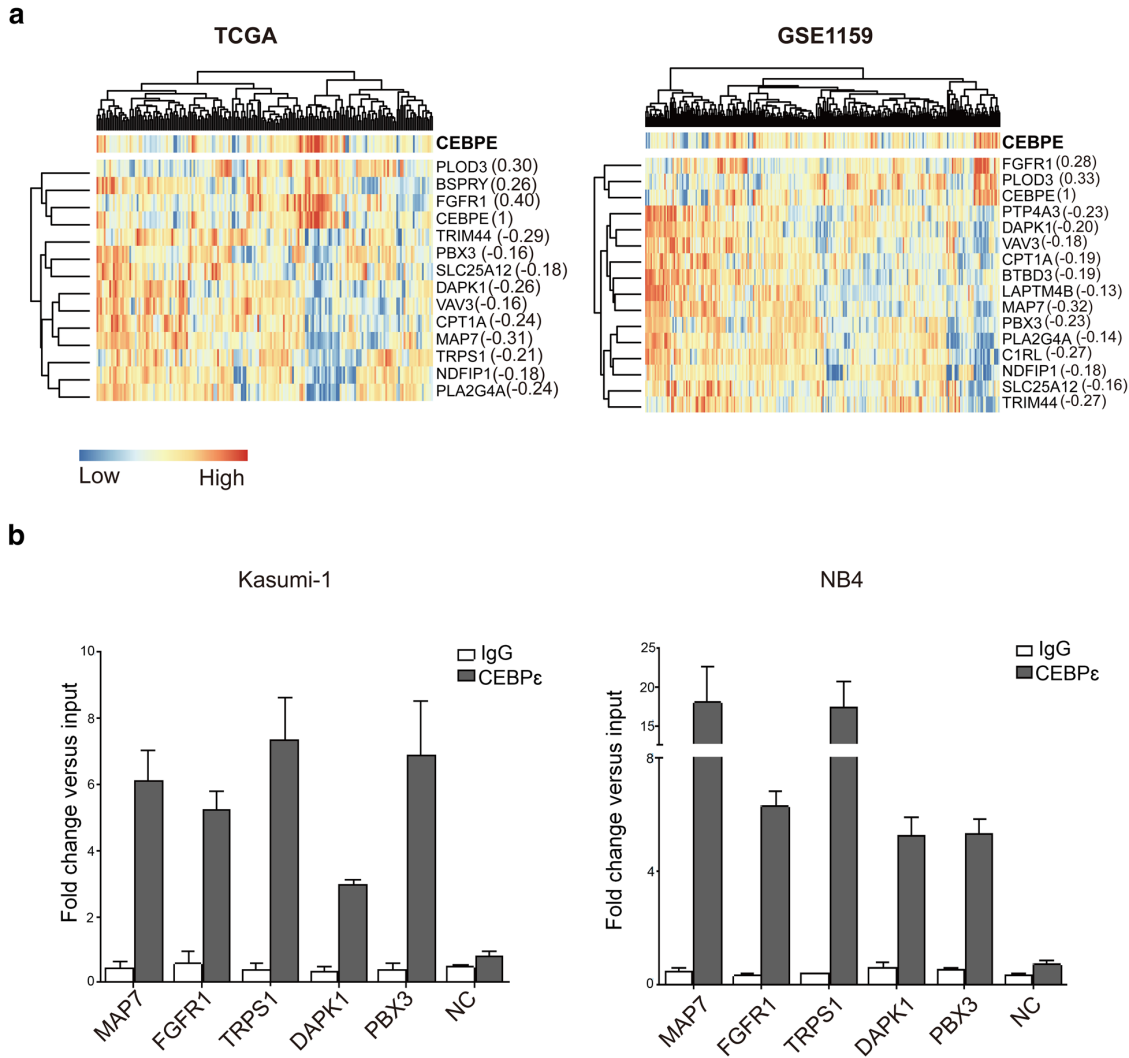
CEBPE regulates known predictors of AML. **a** Heatmaps for gene expression of CEBPE and known prognostic factors of AML in TCGA and GSE1159 datasets. The Pearson correlation coefficient between expression values of CEBPE and each known predictor was listed in the brackets. **b** ChIP-qPCR was performed using the anti-CEBPE in Kasumi-1 and NB4 cell lines. Data are shown as fold enrichment of ChIPed DNA vs. input DNA. Error bars represent SD of triplicate measurements. NC: negative control

According to the above results, we showed that CEBPE expression was an independent prognostic factor for AML survival, relapse and allogeneic transplantation. Then, we attempted to explain the molecular mechanism of favorable outcome induced by increase of CEBPE expression. An international collaborative study reported by Li et al. [28] identified a 24-gene prognostic signature based on the data analyses of 1324 AML patients, and improved the established risk classification of AML prognosis. The identified 24 genes were ALS2CR8, ANGEL1, ARL6IP5, BSPRY, BTBD3, C1RL, CPT1A, DAPK1, ETFB, FGFR1, HEATR6, LAPTM4B, MAP7, NDFIP1, PBX3, PLA2G4A, PLOD3, PTP4A3, SLC25A12, SLC2A5, TMEM159, TRIM44, TRPS1, and VAV3, the increased expression levels of which were significantly associated with worse (22 genes) or favorable (two genes: FGFR1 and PLOD3) OS of AML. We found that CEBPE expression was significantly correlated with these known predictors of AML. As many as 13 genes were co-expressed with CEBPE in TCGA dataset (P-value < 0.05, Fig. 6a left panel), and 15 genes were co-expressed with CEBPE in GSE1159 dataset (P-value < 0.05, Fig. 6a right panel). Interestingly, CEBPE expression level was positively correlated with FGFR1 and PLOD3 in both datasets, which were reported as favorable factors, while negatively correlated with other genes which reported as predictors for poor outcome. This observation was consistent with our results that high expression of CEBPE predicted longer survival and lower relapse rate. Given the fact that CEBPE was an important transcription factor in regulating myeloid differentiation [29, 30], we hypothesized that CEBPE might regulate the expression of these known prognostic factors by localizing on their promoters, and verified using ChIP-qPCR assay in NB4 and Kasumi-1 cells. The results showed that CEBPE actually occupied on the promoters of known predictors, suggesting the regulation role of CEBPE on genes associated with AML prognosis.

## Discussion

In the clinical setting, it is important to identify prognostic factors to direct the appropriate treatments and predict outcomes. Patients with a molecular profile that is associated with a favorable risk have relatively good outcomes with chemotherapy, whereas patients with an unfavorable-risk profile require allogeneic transplantation during the first remission to improve their prognosis [5, 31]. Based on the analyses of several independent datasets, we identified CEBPE expression as an independent prognostic factors for AML patients. Low-expression of CEBPE was found to be associated with shorter OS, EFS and higher relapse rate, indicating adverse outcome of AML. Importantly, both RNA-Seq and microarray data supported this results, suggesting that CEBPE expression was a reliable prognostic factor. In addition, CEBPE expression was proved to have prognostic significance for wild type patients of various genes, providing useful information for prognosis of patients without molecular alterations. Moreover, CEBPE expression was also a potential prognostic factor for allogeneic transplantation. This observation could be easily used in routine clinical practice, as CEBPE expression could be tested before deciding if allogeneic transplantation should be implemented, and allogeneic transplantation surgery would be recommended only for CEBPE low-expressed patients, which will provide accurate information for therapeutic decisions.

The generation and development of AML are associated with the disregulation of various transcription factors (TFs) [32]. Especially, the abnormal expression of TFs which are important in hematopoietic or myeloid differentiations would lead to the accumulation of myeloblasts in the bone marrow and peripheral blood [33]. Previous studies suggested that CEBPE was indispensable for myeloid normal differentiation progress [30, 34]. For example, CEBPE knockout mice die within a few months of birth due to the loss of mature neutrophils or eosinophils [35]. Similarly, patients with a frame-shift mutation in CEBPE are suffered from specific granulocyte deficiency disease [36]. These observations imply that CEBPE may play a pivotal role in granulocytic maturation and exert an important function in myeloid differentiation. Our observations suggested that CEBPE localized on the promoters of a series of known predictors of AML survival, and had positive or negative co-expression relationship with these target genes. This result highlighted the reasons of why CEBPE expression showed significant prognostic power. Importantly, it is much more practical and economical to test the expression of one driver gene (CEBPE) than to test several passenger genes. Therefore, CEBPE expression holds great potential for clinical application in risk stratification and outcome prediction in AML.

## Conclusion

Our findings indicated that CEBPE expression was an independent prognostic factor for AML survival, relapse and allogeneic transplantation, which will provide useful information for outcome prediction and therapeutic decisions.

## Additional file

**Additional file 1: Table S1.** qPCR primer sequences.

## Data Availability

The datasets analyzed in the current study are available in The Cancer Genome Atlas (TCGA) (http://cancergenome.nih.gov/) and Gene Expression Omnibus (GEO) (https://www.ncbi.nlm.nih.gov/geo/).

## Abbreviations

AML: acute myeloid leukemia
OS: overall survival
EFS: event-free survival
ChIP: chromatin immunoprecipitation
WBC: white blood cell
PB: peripheral blood
BM: bone marrow
HR: hazard ratios
KNN: k-Nearest Neighbor
TFs: transcription factors
ELN: European LeukemiaNet
ITD: internal tandem duplication
CI: confidence interval
