# Supplementary material for: CEBPE expression is an independent prognostic factor for acute myeloid leukemia
Source: J Transl Med. 2019 Jun 4;17:188. doi: 10.1186/s12967-019-1944-x (PMC6549322; doi:10.1186/s12967-019-1944-x)
Supplement: Supplementary file 1 — Additional file 1: Table S1. qPCR primer sequences. [file 12967_2019_1944_MOESM1_ESM.docx]

**Additional file 1: Table S1. qPCR primer sequences**

| Gene | Primer sequences（5'-3') |
| --- | --- |
| MAP7 | F: TCATCATGCCCTACAAAGCTG |
|  | R: TGCCAGATGTGAGGAAGAGTA |
| FGFR1 | F: CCCGTAGCTCCATATTGGACA |
|  | R: TTTGCCATTTTTCAACCAGCG |
| TRPS1 | F: ATGACACTCCTGTTGGGTACT |
|  | R: CGTGCTGCTTGCCATAATGTT |
| DAPK1 | F: GAGTTTGTCGCTCCTGAGATAGT |
|  | R: GCTTAGTGTCTCCAAGAAATGGG |
| PBX3 | F: ACCTCCCAAATTCTGGGGACA |
|  | R: ATCCACCTGTGACTGCACATT |
| NC | F: ACAACTTTGGTATCGTGGAAGG |
|  | R: GCCATCACGCCACAGTTTC |

Note: NC, negative control.
